# Synthesis and Biological Activity of Sterol 14α-Demethylase and Sterol C24-Methyltransferase Inhibitors

**DOI:** 10.3390/molecules23071753

**Published:** 2018-07-17

**Authors:** David J. Leaver

**Affiliations:** Department of Biology, Geology and Physical Sciences, Sul Ross State University, Alpine, TX 79832, USA; david.leaver@sulross.edu; Tel.: +1-432-837-8115

**Keywords:** sterol biosynthesis, sterol 14α-demethylase, sterol C24-methyltransferase, mechanism-based inactivators, antifungals, azoles, antiparasitic drugs, human African trypanosomiasis, Chagas disease, synthesis

## Abstract

Sterol 14α-demethylase (SDM) is essential for sterol biosynthesis and is the primary molecular target for clinical and agricultural antifungals. SDM has been demonstrated to be a valid drug target for antiprotozoal therapies, and much research has been focused on using SDM inhibitors to treat neglected tropical diseases such as human African trypanosomiasis (HAT), Chagas disease, and leishmaniasis. Sterol C24-methyltransferase (24-SMT) introduces the C24-methyl group of ergosterol and is an enzyme found in pathogenic fungi and protozoa but is absent from animals. This difference in sterol metabolism has the potential to be exploited in the development of selective drugs that specifically target 24-SMT of invasive fungi or protozoa without adversely affecting the human or animal host. The synthesis and biological activity of SDM and 24-SMT inhibitors are reviewed herein.

## 1. Introduction

**Figure 1:**
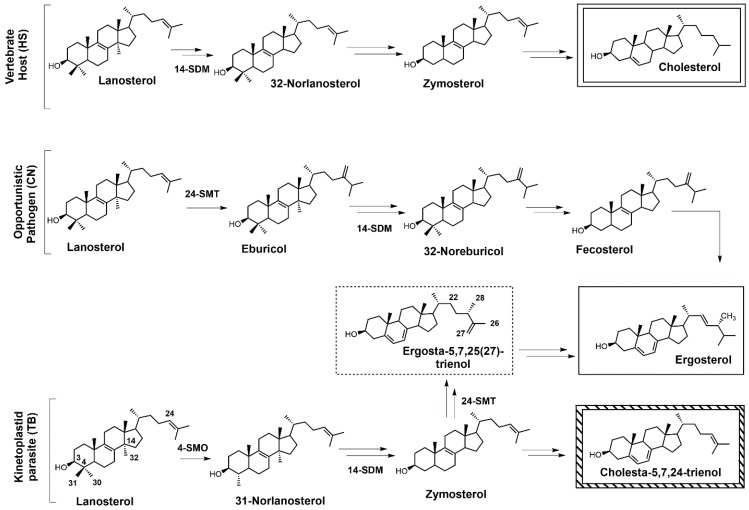
Comparative sterol biosynthesis pathways across kingdoms (adapted from [2]). HS: *Homo sapiens*; CN: *Cryptococcus neoformans*; TB: *Trypanosoma brucei*; 4-SMO: sterol C4-methyl oxidase; 14-SDM: sterol 14α-demethylase; 24-SMT: sterol C24-methyltransferase.

Sterols such as ergosterol and cholesterol are essential lipid molecules, and they perform numerous cellular roles associated with membrane (bulk) and signal (sparking) functions [1,2,3]. Cholesterol is biosynthesized in humans, while ergosterol or other 24-alkylated sterols are biosynthesized in opportunistic fungi and parasitic protozoa (Figure 1) [2,3,4]. This difference in sterol production can be exploited in the development of drugs that are designed to selectively block ergosterol biosynthesis in invasive fungi or protozoan parasites without harming the human host [3,4,5,6,7]. Infectious diseases caused by parasitic protozoa take a heavy toll on human health, are widespread, and are increasing in resistance to current chemotherapies [8]. Leishmaniasis is threatening around 350 million people in more than 98 countries [9], while the World Health Organization (WHO) has estimated that 16–18 million people are infected with Chagas disease [10], and it has been suggested that 70 million people in Africa are at risk of human African trypanosomiasis (HAT; sleeping sickness) [11]. Leishmaniasis is caused by various species of *Leishmania*, while the causative pathogens for Chagas and HAT are *Trypanosoma cruzi* and *Trypanosoma brucei*, respectively. It is well known that these diseases are life-threatening, and without proper treatment they are often fatal. *Leishmania* spp., *T. cruzi*, and *T. brucei* all require ergosterol for growth, and inhibiting the ergosterol biosynthesis pathway in these parasitic protozoa is an ideal approach to treat these infections without harming the human host. It should be noted that fungal infections caused by *Cryptococcus neoformans* have become the leading cause of morbidity and mortality in acquired immune deficiency syndrome (AIDS) patients and other immunocompromised patients, and it is reported that 5–10% of AIDS patients in the United States suffer from these life-threatening infections [12,13].

Selective inhibition of fungal SDM is one of the most common ways to treat fungal infections, and the majority of drugs that target fungal sterol 14α-demethylase (SDM) possess an azole side chain [12,13,14,15,16,17]. For these azole drugs to be efficacious, they need to have greater affinity for fungal SDM versus mammalian SDM [15,16]. Sterol C24-methyltransferase (24-SMT), unlike SDM, is not found in humans but is present in both fungi and protozoa (Figure 1), offering a selective way to inhibit ergosterol biosynthesis [15]. It is important to note that antifungal azoles are heavily used in agriculture [17,18,19], and azole resistance is becoming a major problem [20]. One mechanism of resistance to these antifungal azoles is the activation of efflux pumps that transport azoles out of fungal cells [21]. There is a huge medical need to develop new fungicides and medicinal drugs that specifically block ergosterol biosynthesis and are not likely to develop resistance.

**Figure 2:**
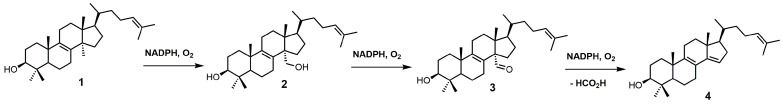
Conversion of lanosterol (**1**) into 4,4-dimethyl-5α-cholesta-8,14,24-trien-3β-ol (**4**) by sterol 14α-demethylase (SDM).

SDM, also known as P450_14DM_ or CYP51, catalyzes the removal of the C-32 methyl group of lanosterol (**1**) via a repetitive three-step process that uses reduced nicotinamide adenine dinucleotide phosphate (NADPH) and oxygen (Figure 2) to ultimately produce 4,4-dimethyl-5α-cholesta-8,14,24-trien-3β-ol (**4**) [22,23]. SDM transforms the C-32 methyl group of lanosterol into an alcohol (**2**), an aldehyde (**3**), and then formic acid with the insertion of the Δ14–15 double bond (Figure 2) [24]. Each P450 catalytic cycle involves the reduction of heme ferric iron to the ferrous state, the binding of molecular oxygen, and subsequent protonation to form a ferric hydroperoxo intermediate [24]. Further protonation of the distal oxygen atom of the ferric hydroperoxo intermediate causes heterolytic scission of the O–O bond, resulting in the loss of water and the formation of an Fe^4+^ oxo porphyrin cation radical, which is the catalytically active species [24]. Once the substrate is oxygenated, the iron returns to its ferric state ready for another catalytic cycle [24].

## 2. Sterol SDM Inhibitors

**Figure 3:**
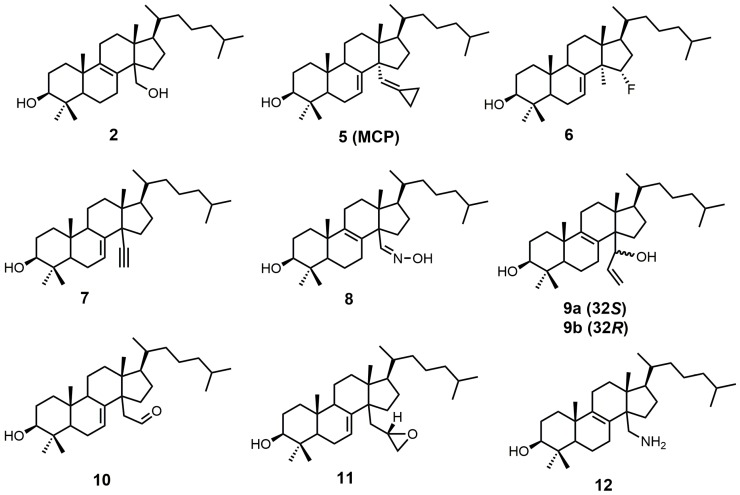
Sterol-based inhibitors of sterol 14α-demethylase (SDM).

Sterol-based SDM inhibitors have been reported in the literature [22,23,24,25,26,27,28,29,30] (Figure 3); however, they are not as commonly reported as azoles [12,17,31,32,33,34,35,36,37,38,39,40,41,42,43,44,45,46]. This is likely due to the limited number of functional groups on lanosterol that can be synthetically modified [47], in addition to the difficult and time-consuming syntheses involved with sterol functional-group manipulation. The preferred substrate of SDM is compound **2**, which is the most potent natural inhibitor of SDM [25]. Compound **2** has a half-maximal inhibitory concentration (IC_50_) value of 7.8 μM against SDM, and it can be made synthetically in nine steps starting from lanosterol [25,48].

14α-Methylenecyclopropyl-Δ7-24,25-dihydrolanosterol (**5** or MCP) was observed to be a competitive inhibitor of F105-containing *T. brucei* CYP51 (*Tb*CYP51)and *Lesihmania infantum* orthologos, while for *T. cruzi,* MCP was presumed to act as a mechanism-based inhibitor (suicide substrate) [24]. The cyclopropyl ring of MCP is presumably opened as MCP binds to *T. cruzi* CYP51 (*Tc*CYP51), forming a covalent bond with the prosthetic heme group [24]. The crystal structure of *Tb*CYP51 covalently bound with MCP has been reported [24]. Despite MCP having the same *K*_d_ values of 0.5 μM for both *Tb*CYP51 and *Tc*CYP51, the observed half-maximal effective concentration (EC_50_) of MCP against *T. brucei* was >50 μM, while *T. cruzi* cell growth was inhibited by 50% at a MCP concentration of 3 μM [49]. MCP inhibits *Tc*CYP51 more than *Tb*CYP51, which is likely due to MCP acting as a suicide substrate for *Tc*CYP51 and as a competitive inhibitor for *Tb*CYP51 [24].

**Figure 4:**
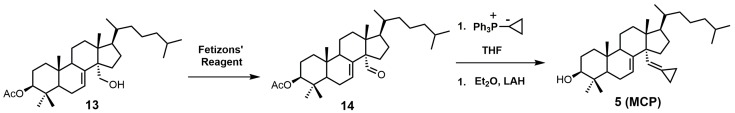
The synthesis of 14α-methylenecyclopropyl-Δ7-24,25-dihydrolanosterol (MCP) (**5**).

MCP can be synthesized in three steps starting with 3β-acetyloxylanost-7-en-30-ol (**13**) (Figure 4) [24]. Compound **13** can be synthesized directly from lanosterol in 12 steps [29,48]. Oxidation of compound **13** with Fetizon’s reagent produced 3β-acetyloxylanost-7-en-30-al (**14**), which was then converted into MCP (**5**) via a Wittig reaction using a cyclopropyltriphenylphosphium ylide, followed by acetyl deprotection with lithium aluminum hydride (LAH) [24].

**Figure 5:**
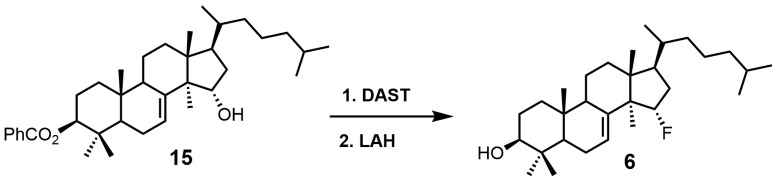
The synthesis of 15α-fluoroIanost-7-en-3β-ol (**6**).

15α-Fluorolanost-7-en-3β-ol (**6**) was noted to be a weak competitive inhibitor of SDM with a *K_i_* value of 315 μM [28]. Metabolic studies have indicated that compound **6** is converted to 15α-fluoro-3β-hydroxylanost-7-en-32-al by hepatic microsomal SDM and that the 15α-fluoro substitution blocks further metabolic conversion into other cholesterol biosynthetic intermediates [28]. The starting material used to synthesize compound **6** was 3β-benzoyloxy-lanost-7-en-15α-ol (**15**) (Figure 5) [50,51]. Compound **15** was reacted with diethylaminosulfur trifluoride (DAST) to install the fluorine at C-15, and the benozyl protecting group was removed by LAH [28].

**Figure 6:**
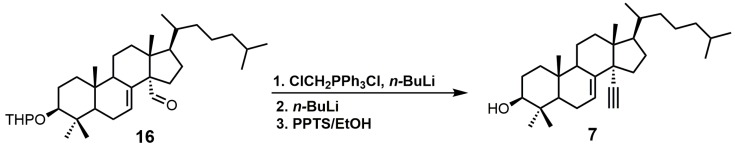
The synthesis of 4,4-dimethyl-14α-ethynylcholest-7-en-30-ol (**7**).

4,4-Dimethyl-14α-ethynylcholest-7-en-30-ol (**7**) was observed to have *K*_d_ values of 1.2 μM against *Tb*CYP51 and 1.3 μM against *Tc*CYP51 [49]. Compound **7** had a weaker affinity for both *Tb*CYP51 and *Tc*CYP51 in comparison to MCP. Compound **16** (Figure 6) is the starting material required to synthesize compound **7**, and compound **16** can be synthesized in 11 steps starting from lanosterol [23]. Aldehyde **16** was reacted with the ylide of chloromethyltriphenylphosphonium chloride, followed by the addition of *n*-butyllithium to introduce the desired alkyne functionality (Figure 6) [29]. The tetrahydropyran (THP) protecting group was removed by the use of pyridinium *p*-toluenesulfonate (PPTS) in ethanol to yield compound **7** [29].

**Figure 7:**
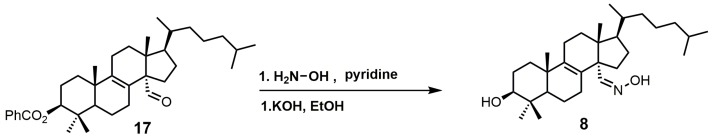
The synthesis of lanost-8-en-32-alkoxime-3β-ol (**8**).

Lanost-8-en-32-alkoxime-3β-ol (**8**) was reported as having an IC_50_ value of 1.1 μM against SDM [25]. Compound **8** was readily prepared from 3β-benzoyloxy-lanost-8-en-32-al (**17**) in two steps (Figure 7) [52]. The aldehyde functional group of compound **17** was converted into an oxime by the use of hydroxylamine hydrochloride in pyridine [52]. The benzoyl protecting group was removed using potassium hydroxide in ethanol to yield compound **8** [52].

**Figure 8:**
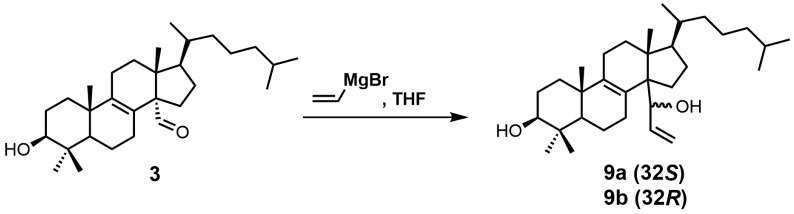
The synthesis of 4,4-dimethyl-14α-(1′hydroxy-2′-vinyl)-5α-cholest-8-en-3β-ols.

A stereochemical preference of the 32-vinyl alcohols (compounds **9a** and **9b**; Figure 8) was observed against SDM [25]. The 32*S*-isomer (compound **9a**) was observed to have an IC_50_ value of 0.75 μM against SDM in comparison to the 32*R*-isomer (compound **9b**), which has a reported IC_50_ value of 3.20 μM [25]. This result illustrates the importance of having optimized stereochemistry in SDM inhibitors. Compounds **9a** and **9b** were readily prepared from lanost-8-en-32-al-3β-ol (**3**) via a Grignard reaction using vinyl magnesium bromide in THF [52]. The Grignard reaction was compatible without needing a protecting group for the 3β alcohol. The two diastereoisomers **9a** and **9b** were successfully separated by medium pressure liquid chromatography (MPLC) [52].

**Figure 9:**
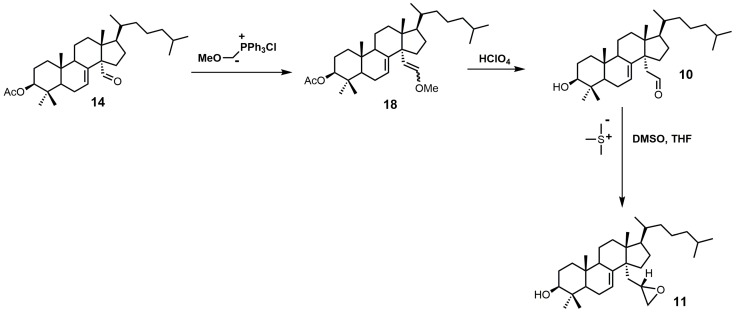
The synthesis of 32*S*-oxiranyllanost-7-en-3β-ol (**11**).

Aldehyde **10** and epoxide **11** (Figure 9) were both observed to inhibit total cholesterol and lathosterol biosynthesis by >89% at an inhibitor concentration of 10 μM, and these compounds were believed to inhibit SDM [27]. Compounds **10** and **11** have *K_i_* values of 3 and 0.61 μM, respectively, while the 32*R*-oxiranyllanost-7-en-3β-ol isomer has a *K_i_* value of 2 μM [27]. The synthesis of 32*S*-oxiranyllanost-7-en-3β-ol (**11**) and the 32*R* isomer started with a Wittig reaction between aldehyde **14** and the ylide of (methoxymethyl)triphenylphosphonium chloride to yield compound **18** (Figure 9) [22]. Cleavage of the methyl enol ether of compound **18** was achieved by the use of perchloric acid to yield aldehyde **10** [22]. Corey–Chaykovsky reaction conditions were then used to transform compound **10** into compound **11** [22]. A 6:1 diastereomeric mixture of 32*S*-oxiranyllanost-7-en-3β-ol (**11**) and 32*R*-oxiranyllanost-7-en-3β-ol was obtained, and this mixture was successfully purified by high-performance liquid chromatography (HPLC) [22].

**Figure 10:**
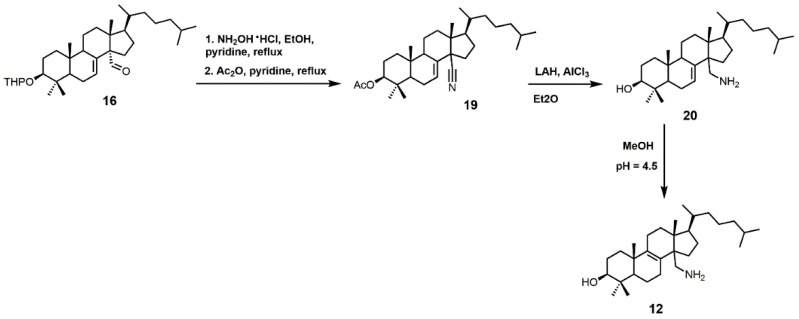
The synthesis of 4,4-dimethyl-14α-aminomethyl-cholest-8-en-3β-ol (**12**).

4,4-Dimethyl-14α-aminomethyl-cholest-8-en-3β-ol (**12**) and 4,4-dimethyl-14α-aminomethyl-cholest-7-en-3β-ol (**20**) were observed to be active against *T. cruzi* SDM with apparent *K*_d_ values of 5.1 and 1.3 μM, respectively [30]. These two amino derivatives have around a 3-fold stronger inhibitory effect on *Candida albicans* SDM in comparison to *T. cruzi* SDM and produce IC_50_ values of around 4 μM against C. *albicans* growth [30]. 4,4-Dimethyl-14α-aminomethyl-cholest-8-en-3β-ol (**12**) can be synthesized starting with compound **16** (Figure 10) [30]. The aldehyde functional group of compound **16** was converted into an oxime with hydroxylamine hydrochloride, which in turn was transformed into nitrile **19** with acetic anhydride and pyridine [30]. Nitrile **19** was then reduced to a primary amine with lithium aluminum hydride and aluminum trichloride to yield compound **20**, which was easily isomerized into compound **12** with acidic methanol [30].

## 3. Azole SDM Inhibitors

**Figure 11:**
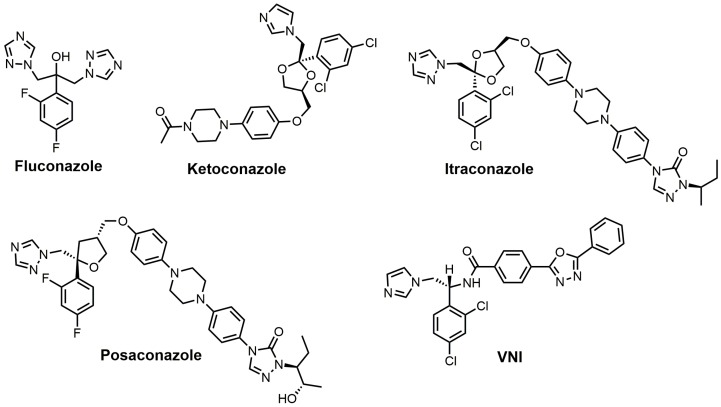
Azole inhibitors of sterol 14α-demethylase (SDM).

Azoles are the largest class of SDM inhibitors, and this group of inhibitors is continuously expanding with the creation of new drugs or molecules with drug-like properties. 1,2,4-Triazole fungicides such as difenoconazole (Score^®^ (Syngenta, Basel, Switzerland)), epoxiconazole (Opal^®^ (TRC, North York, ON, Canada)), flusilazole (Punch^®^ (DuPunt, Wilmington, DE, USA)), and so forth are well-known SDM inhibitors used against agricultural relevant fungal diseases, including powdery mildews, rusts, and leaf-spotting fungi from Ascomycetes and Basidiomycetes [17]. Human fungal infections have been treated with antifungal azoles for a long period of time; chlormidazole was the first azole drug, introduced in 1958 for the treatment of topical mycosis [53]. The older antifungal azoles that were predominately discovered in the 1950–1960s have undergone numerous structural modifications to yield the next generation of antifungal azole drugs. In addition, many of these older antifungal azole drugs have reemerged or undergone structural modifications to be used as potential anti-trypanosomiasis drugs. The renaissance of using old antifungal agents for treating or attempting to treat trypanosomiasis was largely driven by large pharmaceutical companies not prepared to invest heavily in neglected diseases that are prevalent in developing countries where there would be no chance of cost recovery [53,54]. Some of the classic azoles used as standards for fungal SDM inhibition include ketoconazole, fluconazole, itraconazole, and posaconazole, and structures of these drugs are shown in Figure 11. Fluconazole (Diflucan^®^) was released by Pfizer Canada Inc, Kirkland, QC, Canada and was active against *Candida* spp., while itraconazole (Sporanox^®^) was released from Janssen Pharmaceutica in the early 1990s and was active against both *Candida* spp. and *Aspergillus* spp. [17]. Itraconazole from Janssen Pharmaceutica, Beerse, Belgium was approved for use in 1992, while posaconazole (Noxafil^®^) from the Schering-Plough Research Institute, Kenilworth, New Jersey, USA was approved by the food and drug administration (FDA) in 2006 [53]. Posaconazole was noted to be more effective than amphotericin B in the treatment of *Aspergillus* spp. infections, and it is structurally similar to itraconazole (Figure 11) [53]. (*R*)-*N*-(1-(2,4-Dichlorophenyl)-2-(1*H*-imidazol-1-yl)ethyl)-4-(5-phenyl-1,3,4-oxadi-azol-2-yl)-benzamide (VNI) (Figure 11), a new-generation imidazole, has been shown to exert a curative effect for both acute and chronic Chagas disease in a murine model with 100% survival and no observable side effects [14,55]. VNI is available at a low cost (<0.10/mg) and has good oral bioavailability, low toxicity, and favorable pharmacokinetics, which makes this compound an attractive candidate for clinical trials to treat patients with Chagas disease [55].

Fluconazole is a water-soluble, well-tolerated, and cheap first-generation antifungal drug that penetrates the blood–brain barrier [21]. Fluconazole has reported minimum inhibitory concentrations (MIC_90_) required to inhibit the growth of various *Candida* spp. isolates by 90% ranging from 2 to 64 mg/mL [56]. Fluconazole was observed to maintain an in vivo effective dose for 50% (ED_50_) of the murine population with <1.0 mg/kg for 5 days when orally administered in a murine candidiasis model, making it 50 times more potent than ketoconazole [57]. The plasma half-life for fluconazole was 6.1 h, and 75% of the drug was excreted in urine with no changes to its structure [12]. The 2,4-difluorophenyl side was chosen because it was the only phenyl analog that was water-soluble (8 mg/mL), which was needed to enable it to be formulated for intravenous administration. Fluconazole went through safety studies and passed evaluation in humans, where it was shown to very successful in the treatment of *C. albicans* and *C. neoformans* infections but was not as effective against *Aspergillus* infections when compared with itraconazole [12]. Fluconazole has a reported *K*_d_ value of 0.23 μM against *T. cruzi* SDM and an EC_50_ value of approximately 40 μM against *T. cruzi*, while fluconazole has a *K*_d_ value of 0.34 μM for *T. brucei* SDM [21,58]. Fluconazole has an in vitro IC_50_ value of 8 μM against *T. cruzi,* and a crystal structure of *T. cruzi* SDM cocrystallized with fluconazole has been reported [21,59,60,61]. A 6 week oral course of fluconazole was shown to be safe and useful for treating leishmaniasis caused by *Leishmania major* [62].

**Figure 12:**
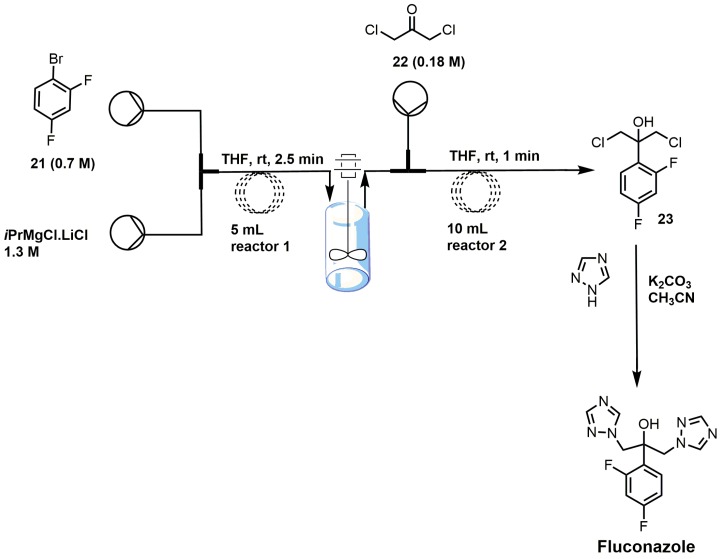
Synthesis of fluconazole using flow-injection methodology (adapted from [39]).

There are several different ways to synthesize fluconazole. One of the popular routes is the oxirane ring opening of 1-[[2-(2,4-difluorophenyl)oxiranyl]methyl]-1*H*-1,2,4-triazole with 1,2,4-triazole and potassium carbonate [17]. One of the more recent syntheses of fluconazole uses a semi-continuous flow method (Figure 12) [39]. 2,4-Difluorobromobenzene (**21**) was reacted with isopropyl magnesium chloride in THF to form a Grignard reagent that was reacted under flow conditions with 1,3-dichloroacetone (**22**) to yield 2-(2,4-difluorophenyl)-1,3-dichloro-2-propanol (**23**) [39]. The synthesis of fluconazole was completed with the nucleophilic attack of intermediate **23** with two 1,2,4-triazoles without using flow chemistry [39].

**Figure 13:**
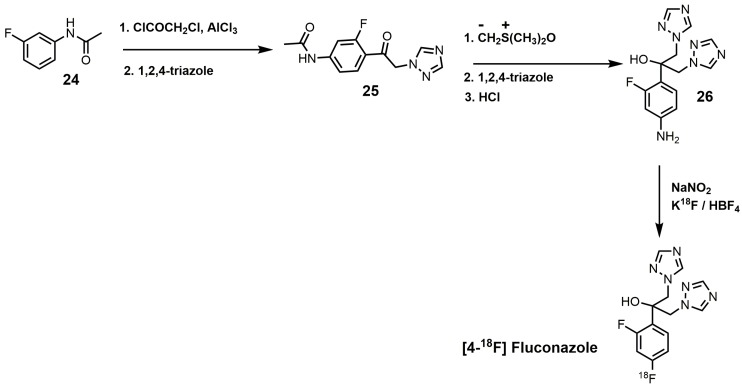
Synthesis of [4-^18^F]fluconazole.

An interesting synthesis of fluconazole is the ^18^F-labeled synthesis of [4-^18^F]fluconazole (Figure 13) [38]. Under Friedyl–Crafts acylation conditions, *m*-fluoroacetanilide (**24**) was reacted with chloroacetylchloride to yield *N*-[4-(2-chloroacetyl)-3-fluorophenyl]acetamide, which was reacted with 1,2,4-triazole to produce compound **25** (Figure 13) [38]. Corey–Chaykovsky conditions were used to convert intermediate **25** into an epoxide that was ring-opened by 1,2,4-triazole, and the *N*-acetyl group was removed by acid hydrolysis to form compound **26**. [4-^18^F]Fluconazole was obtained by a modified Schiemann reaction using 25% tetrafluoroboric acid, sodium nitrite, and potassium [^18^F]-fluoride [38]. [4-^18^F]Fluconazole was synthesized to measure the pharmacokinetics of fluconazole in rats by radioactive measurements of excised tissues and in rabbits by positron emission tomography (PET) [38]. A uniform distribution of radioactivity in most organs was observed in both species upon equilibration of [4-^18^F]fluconazole [38].

Ketoconazole developed by Janssen Pharmaceutica was approved by the FDA in 1981 and was the first broad-spectrum antifungal agent with oral activity; its reported MIC_90_ values range from 0.06 to 4 μg/mL against various *Candida spp*. [12,53]. Ketoconazole is highly active against *Trichophyton mentagrophytes* at 0.1 μg/mL, while *Trichophyton rubrum* and *C. neoformans* are inhibited at 1 μg/mL [12]. Ketoconazole has been shown to prolong the survival of mice infected with *T. cruzi*; however, curing effects were not observed [63,64]. Ketoconazole has a reported *K*_d_ value of 4.4 μM against cloned *Tb*CYP51 [65]. Ketoconazole at a concentration of 0.1 μM in conjunction with 24(*R,S*)-25-epiminolanosterol (EL) at a concentration of 0.3 μM exerts strong antiproliferative effects on *T. cruzi* epimastigotes, leading to >80% inhibition of growth [66].

**Figure 14:**
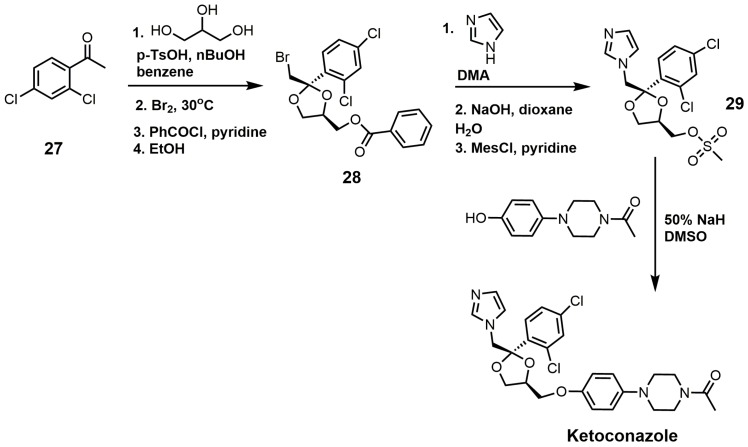
Synthesis of ketoconazole.

The synthesis of ketoconazole (Figure 14) started with the ketalization of 3′,5′-dichloroacetophenone (**27**) with glycerine, and the crude product was brominated [31]. Benzylation of the resulting primary alcohol produced a mixture of *cis*/*trans* isomers, and the desired *cis* isomer (**28**) was obtained by crystallization from ethanol [31]. After the bromine atom was displaced by nucleophilic attack by imidazole, the benzoyl ester was cleaved by sodium hydroxide to produce an alcohol that was activated as a mesylate (**29**). Mesylate **29** was reacted with the sodium salt of 1 acetyl-4-(4-hydroxyphenyl)piperazine to produce ketoconazole [31].

Itraconazole has MIC_90_ values ranging from 0.03 to 64 μg/mL against various *Candida* spp. and a MIC_90_ value of 2.7 μg/mL against *Malassezia furfur* [12]. Itraconazole was noted to have potent in vitro activity against *C. neoformans* [67]. Itraconazole was observed to successfully achieve irreversible damage against various fungi, including *C. albicans*, *C. neoformans*, *T. rubrum*, *Paracoccidiodes brasiliensis,* and *Aspergillus fumigatus* [12,68]. Itraconazole absorption is enhanced by food intake, and oral solutions provide higher serum concentrations in comparison to capsules [12,69,70]. Itraconazole is very active in vitro against *Microsporum canis* and other dermatophytes associated with scalp ringworm infections [12]. The selectively index of itraconazole for fungal SDM versus human SDM was reported to be 25, which was much better than the selectively index of ketoconazole [53,71]. Itraconazole was observed to have an ED_50_ value of 1 μM against *T. brucei* parasites, and when combined with 25-azalanosterol (AZAL), parasite death resulted [2]. Itraconazole exhibited in vitro activity against *Leishmania mexicana mexicana* amastigotes in macrophages, and 50% inhibition of ergosterol synthesis was observed at 0.15 μM, which was more potent than that observed for ketoconazole (0.21 μM) [72]. Itraconazole has been reported to successfully treat cutaneous leishmaniasis; however, it was not as effective against *Leishmania braziliensis* [53,73,74,75]. It is noted that itraconazole has not been able to completely eradicate *T. cruzi* from experimentally infected animals or human patients in studies investigating it as a possible treatment for Chagas disease [76].

**Figure 15:**
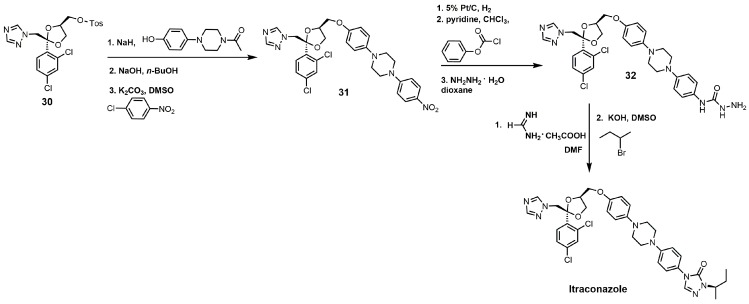
The synthesis of itraconazole.

The synthesis of itraconazole can be started by reacting compound **30** with sodium hydride and 1 acetyl-4-(4-hydroxyphenyl)piperazine to yield *cis*-1-acetyl-4-[4-[[2-(2,4-dichlorophenyl)-2-(1*H*-1,2,4-triazol-1-ylmethyl)-1,3-dioxolan-4-yl]methoxy] phenyl]-piperazine (Figure 15) [34]. The *N*-acetyl group can be removed by sodium hydroxide, and the unprotected piperazine nitrogen under basic conditions with potassium carbonate can undergo a nucleophilic aromatic substitution with *p*-chloronitrobenzene to yield compound **31**. The aromatic nitro group was reduced to an aniline functional group with hydrogen gas and 5% platinum on carbon, and the aniline derivative was converted into a carbamate with phenyl chloroformate [34]. Reacting the carbamate with hydrazine produced the hydrazine carboxamide **32**. Compound **32** was initially treated with formamidine acetate and then with 2-bromobutane in the presence of potassium hydroxide to yield itraconazole (Figure 15) [34].

Posaconazole (Figure 11) has already been registered in the European Union (2005), Australia (2005), and the United States of America (2006) as a prophylactic for invasive fungal infections in addition to azole-resistant candidiasis [77]. Posaconazole was observed to have a MIC value of 20 nM and an IC_50_ value of 14 nM against the epimastigote form of *T. cruzi* and a MIC value of 3 nM and an IC_50_ value of 0.25 nM against the clinically relevant intracellular amastigote form in vitro [78]. Posaconazole demonstrated potent in vivo anti-parasitic activity in a murine model of acute Chagas disease, which was most likely due to its high binding affinity for *Tc*CYP51, good pharmacokinetic properties, and long terminal half-life [77,78]. The crystal structure of posaconazole bound to SDM of *T. brucei* has been solved and provides valuable insights into how posaconazole binds to its target [60]. Posaconazole is active against nitrofuran- and nitroimidazole-resistant *T. cruzi* strains and is able to induce radical parasitological cures in both chronic and acute experimental Chagas disease [76]. An Argentinean woman in Spain who suffered from chronic Chagas disease was successfully treated with posaconazole, although requiring concurrent immunosuppressive therapy [79]. The woman was initially treated with benznidazole, which only resulted in a reduction of *T. cruzi* levels and not an eradication as was achieved with the use of posaconazole [79]. The major drawback of posanconazole is that it is very expensive to produce (>$1000/patient) as it is very low yielding (<1% overall), which will limit its widespread use in developing countries even if it is successful in obtaining approval for treating Chagas disease [55,77,79].

**Figure 16:**
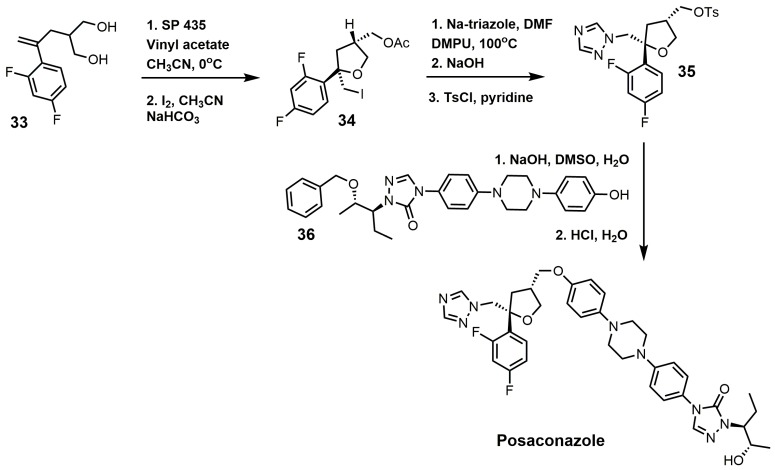
Synthesis of posaconazole.

One of the well-established synthetic routes for the synthesis of posaconazole starts with the enzymatic desymmetrization of the homochiral diol (**33**) with hydrolase Novo SP 435 and vinyl acetate, followed by iodocyclization to produce compound **34** (Figure 16) [37,80]. It should be noted that 169 hydrolases were screened in order to make the first step a practical synthetic route [37]. A 1,2,4-triazole displaced the iodine in compound **34**, and the acetate group was replaced with a tosylate to yield compound **35**. Compound **35** was then reacted with compound **36** under basic conditions, and the benzyl group was removed under acidic conditions to yield posaconazole [81,82]. 

VNI (Figure 11) is a potent inhibitor of *T. cruzi* SDM and is nontoxic and highly selective [55]. Mice infected with an acute or chronic *T. cruzi* infection were treated orally with VNI at 25 mg/kg for 30 days, which resulted in 100% survival of the infected mice [55]. VNI cocrystallized with both *T. cruzi* and *T. brucei* SDM has been reported [21,83]. The *K*_d_ value for VNI against *T. brucei* SDM is 0.37 μM, and it is 0.09 μM against *T. cruz*i SDM [14,83]. VNI exhibits low general cytotoxicity and a potent cellular effect on *T. cruzi* amastigotes, with an EC_50_ value of 1.2 nM, in addition to an excellent selectivity index (human/*T. cruzi* of >50,000) [14]. An advantage of VNI, unlike posaconazole or fluconazole, is that it does not induce *T. cruzi* gene expression, which means it is not as likely to cause drug resistance [14]. As mentioned earlier, VNI is available at a low cost (<$0.10/mg), which makes this compound an attractive candidate for clinical trials [55]. A 2-fluoro-4-(2,2,2-trifluoroethoxy)phenyl derivative of VNI was recently reported to inhibit *C. albicans* and *A. fumigatus* SDM by 89% and 65%, respectively [46]. This 2-fluoro-4-(2,2,2-trifluoroethoxy)phenyl derivative of VNI was observed to have a *K*_d_ value of 10 nM against *C. albican*s SDM and of 20 nM against *A. fumigatus*, while posaconazole had a *K*_d_ value of 81 nM against *C. albican*s SDM and of 131 nM against *A. fumigatus* [46]. A crystal structure of *A. fumigataus* SDM cocrystallized with this VNI derivative was obtained [46].

**Figure 17:**
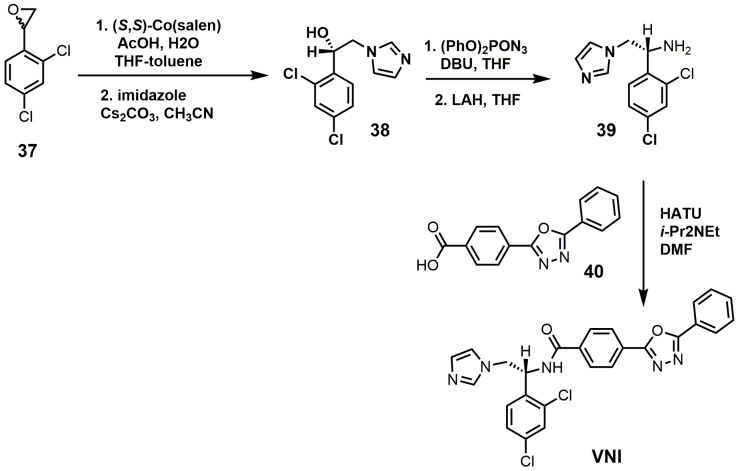
Synthesis of (*R*)-*N*-(1-(2,4-dichlorophenyl)-2-(1*H*-imidazol-1-yl)ethyl)-4-(5-phenyl-1,3,4-oxadi-azol-2-yl)-benzamide (VNI).

VNI can be synthesized in five steps that are scalable to multi-gram quantities with >98% purity as needed for in vivo animal studies (Figure 17) [84]. The racemic epoxide (**37**) was resolved with (*S*,*S*)-Co(salen) and then opened with imidazole under basic conditions to yield compound **38** [84]. Compound **38** was reacted with diphenylphosphoryl azide to replace the secondary alcohol with an azide group and was subsequently reduced with lithium aluminum hydride to yield amine **39** [84]. VNI was obtained by reacting compounds **39** and **40** under amide bond coupling conditions with 1-[bis(dimethylamino)methylene]-1*H*-1,2,3-triazolo[4,5-b]pyridinium 3-oxide hexafluoro-phosphate (HATU) [84].

## 4. 24-SMT Inhibitors

**Figure 18:**
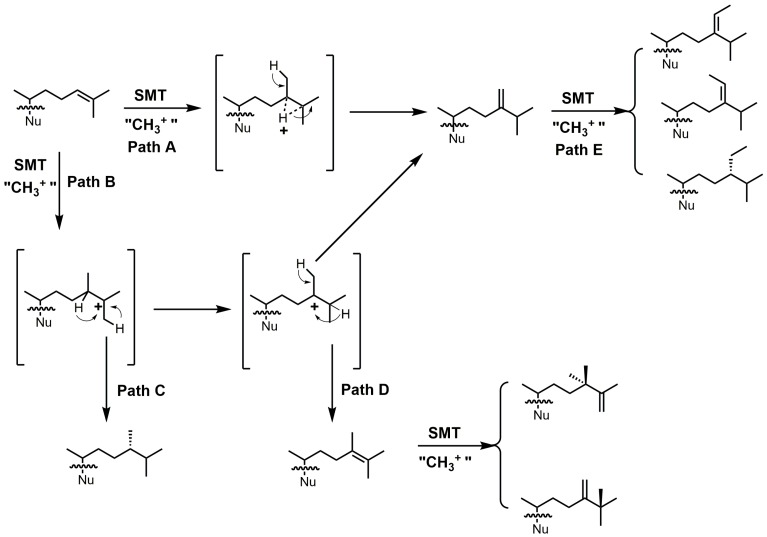
Different C24-alkylation pathways for sterol C24-methyltransferase (24-SMT) substrates (adapted from [4]). Nu: sterol nucleus.

24-SMT catalyzes a methylation–deprotonation reaction that involves electrophilic alkylations of a double bond at C-24 by a methyl cation originating from *S*-adenosyl methionine (SAM) (Figure 18) [4]. The majority of characterized 24-SMTs are multifunctional and possess a very high substrate specificity, often yielding only a single C-24 methylated olefin product; however, there are a few atypical 24-SMTs that convert substrates to a variety of 24-alkyl(idene) products including *T. brucei* SMT1 and *Glycine max* SMT1 [4]. The various pathways possible with 24-SMT catalysis are outlined in Figure 18. 

**Figure 19:**
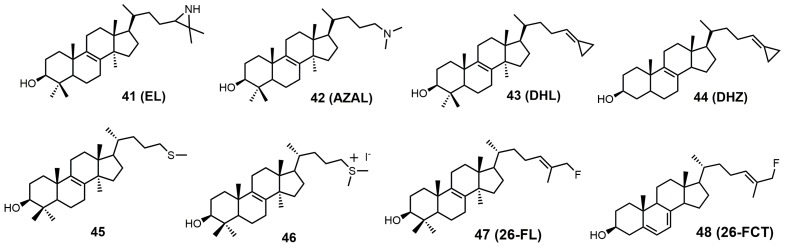
Structures of sterol C24-methyltransferase (24-SMT) sterol-based inhibitors.

The structures of 24-SMT inhibitors typically have a modified lanosterol side chain (Figure 19).

**Figure 20:**
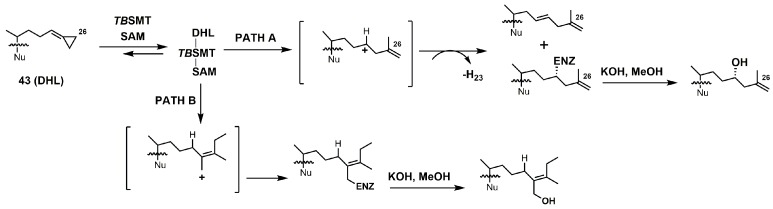
Proposed inhibitory mechanism of *Tb*SMT with 26,27-dehydrolanosterol (DHL) (adapted from Miller et al., 2017). Nu: lanosterol nucleus.

Most of the inhibitors outlined in Figure 19 are suicide/irreversible inhibitors that have an electron withdrawing group strategically placed at or near position 25 on the sterol side. The cyclopropylidine derivatives (**43**–**44**) are spring-loaded electrophiles that are opened irreversibly with nucleophilic attack from 24-SMT (Figure 20).

**Figure 21:**
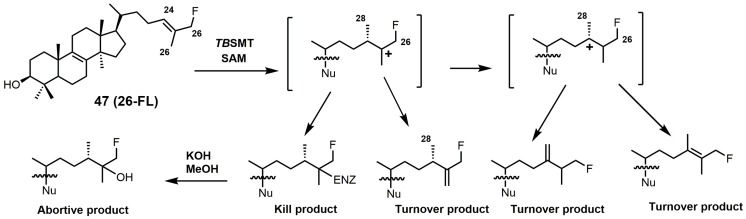
C24-methylation pathway of 26-fluorolanosterol (26-FL) (**47**) with *Tb*SMT. Adapted from [5].

Having a strong electron withdrawing group near the Δ24–25 bond, such as fluorine, affects the intermediate cation formation and timing of the C-24 methylation reaction, promoting partitioning towards irreversible covalent modification over turnover products (Figure 21) [5].

24(*R,S*)-25-Epiminolanosterol (EL; **41**) has a reported IC_50_ value of around 0.3 μM against *C. neoformans* and has comparable activity to itraconazole [6]. EL was observed to be a potent non-competitive inhibitor of 24-SMT from sunflower embryos with a *K_i_* value of 3.0 nM, while sitosterol and 24(28)-methylene cycloartenol were observed to be competitive inhibitors, with *K_i_* values of 26 and 14 μM, respectively [85]. EL as 2-tritio-24(*R*,*S*)-25-epiminolanosterol was reported to inhibit 24-SMT of *Gibberella fujikuroi* and was metabolized to 25-aminolanosterol and lanosterol [86]. EL is a potent inhibitor against *C. albicans* 24-SMT with a *K_i_* value of 11 nM and an IC_50_ value of 5 μM [3]. EL added to cultures of rat hepatoma cells (H4) interrupted the conversion of lanosterol to cholesterol [87]. EL caused the accumulation of zymosterol at 45 μM and at 4.5 μM caused the accumulation of desmosterol [87]. H4 rat hepatoma cells seeded into either full growth or lipid-depleted medium containing 22.5 μM EL would not grow unless the media was supplemented with low-density lipoproteins (60 μg/mL) [87].

**Figure 22:**
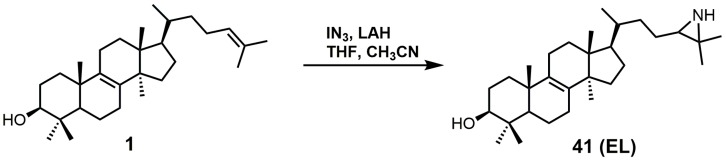
The synthesis of 24(*R,S*),25-epiminolanosterol (EL).

EL was observed to have a *K_i_* value of 49 nM against *T. brucei* 24-SMT [88]. An amastigote form of *T. cruzi* proliferating in a liver infusion tryptose medium was treated with EL, and growth was completely arrested; lysis occurred at an EL concentration of 6 μM [89]. EL was recently reported as an inhibitor of *Acanthamoeba* spp. trophozoite growth with an IC_50_ value of 7 nM, and in this study, EL yielded 20-fold higher inhibition compared to the reference drug voriconazole [90]. EL exhibited tight biding against both 24- and 28-SMT with *K_i_* values of around 9 nM [90]. EL (**41**) can readily be synthesized directly from unprotected lanosterol with an excess of iodine azide and the addition of LAH (Figure 22) [91].

**Figure 23:**
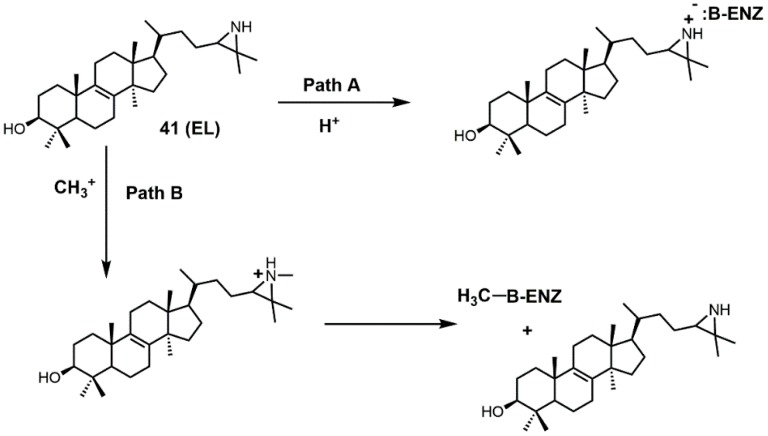
Proposed pathways for inhibition of sterol C24-methyltransferase (24-SMT) with 24(*R,S*)-25-epiminolanosterol (EL) (adapted from [92]).

There are several different ways EL is thought to inhibit 24-SMT (Figure 23). EL could have its aziridine nitrogen atom protonated (path A) and then form an ammonium salt that can electrostatically interact with a polar amino acid in the active site of 24-SMT (such as a carboxylate group) [92]. Alternatively, EL could be methylated by 24-SMT and donate its *N*-methyl group to the active-site residue of 24-SMT, thereby inactivating it [92].

AZAL was reported to have a *K_i_* value of 54 nM and an IC_50_ value of 3 μM against 24-SMT of *C. albicans* [3], while it had a *K_i_* value of 30 nM against 24-SMT from sunflower embryos [3,85]. AZAL was noted to be a potent inhibitor of the ascomycetous fungus *P*. *brasiliensis* (Pb) with an IC_50_ value of 30 nM and a *K_i_* value of 14 nM against Pb 24-SMT [93]. AZAL was observed to inhibit *P. brasiliensis* growth much more than for the related yeasts *Saccharomyces cerevisiae* and *C. albicans* [93]. AZAL has a *K_i_* value of 39 nM against *T. brucei* 24-SMT and an ED_50_ value of 1 μM against *T. brucei* parasites [2,88]. AZAL failed to inhibit cultured human epithelial cells (HEK) with an ED_50_ value of >50 μM and a therapeutic index of 25 [2]. AZAL has a reported IC_50_ value of approximately 1 μM against both the procylic and bloodstream forms of *T. brucei,* and the bloodstream form of *T. brucei* was not rescued with cholesterol absorption from the host, highlighting the importance of ergosterol in cell proliferation of the parasite [94]. Combing AZAL and itraconazole at ED_50_ concentrations to the bloodstream form of *T. brucei* in lipid-depleted medium resulted in cell death and significant growth inhibition when grown in full growth medium [2].

**Figure 24:**
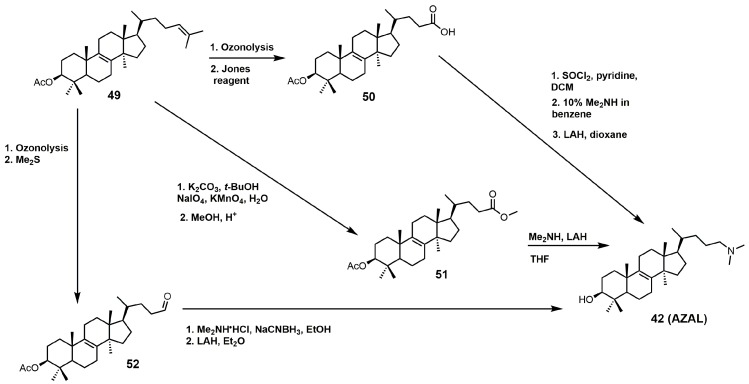
Three synthetic routes used to prepare 25-azalanosterol (AZAL).

AZAL can readily be synthesized via three different synthetic routes, which are outlined in Figure 24 [4,95,96]. All three routes involve converting the Δ24–25 double bond of lanosterol into an activated carbonyl group (aldehyde, carboxylic acid, or ester) followed by nucleophilic attack with dimethylamine. Each synthetic route was completed with protecting group removal by LAH.

26,27-Dehydrolanosterol (DHL; **43**) was reported as an inhibitor of *Acanthamoeba* spp. trophozoite growth, with an IC_50_ value of 6 μM making it a much weaker binder in comparison to EL (IC_50_ of 7 nM) [90]. DHL is metabolized to a favorable substrate, which irreversibly inhibits *Tb*-24SMT, and the ED_50_ values of DHL incubated with procyclic or bloodstream forms of *T. brucei* are 10 and 20 μM, respectively [97]. 26,27-Dehydrozymosterol (DHZ) was reported to inhibit 24-SMT from *C. albicans* with a *K_i_* value of 9 μM and a reported *k*_inact_ value of 0.03 min^−1^ [3]. [3-^3^H]26,27-Dehydrozymosterol was noted to inhibit 24-SMT from *S. cerevisiae* with an apparent *K_i_* value of 1.1 μM [98]. DHZ also inhibits SMT2-2 of *Glycine max* (soybean) with a *K_i_* value of 9.3 μM and a *K*_inact_ value of 0.023 min^−1^ [99], while DHZ irreversibly inhibits *Arabidopsis* SMT2 with a *K_i_* value of 49 μM and a *k*_inact_ value of 0.009 s^−1^ [100]. *T. brucei* 24-SMT was inhibited by DHZ with a *K_i_* value of 29 μM and a *k*_inact_ value of 0.26 min^−1^. Against *T. brucei* 24-SMT, DHZ was noted to be a weaker inhibitor in comparison to EI and AZAL [88]. DHL cannot inhibit *T. brucei* 24-SMT or yeast 24-SMT because “uncharged” 4,4-dimethylsterols cannot bind productively to these types of SMT [88]. 26,27-Dehydrocycloartenol (DHC; **53**) was observed to be a substrate for soybean SMT with *K*_m_ and *k*_cat_ values of 10 mM and 0.018, respectively [101]. There are two main pathways along which DHL can irreversibly inhibit *Tb*SMT (Figure 20) [97]. Path A has a carbocation intermediate on carbon 24, while path A has the intermediate on carbon 27, and both carbocations are reactive with SMT. Either biosynthetic route results in the metabolized DHL to irreversibly bind to *Tb*SMT, and the resulting prosthetic group was hydrolyzed with potassium hydroxide in methanol to yield the C-24 or C-27 alcohol (Figure 23) that was characterized by gas chromatography–mass spectrometry (GCMS).

**Figure 25:**
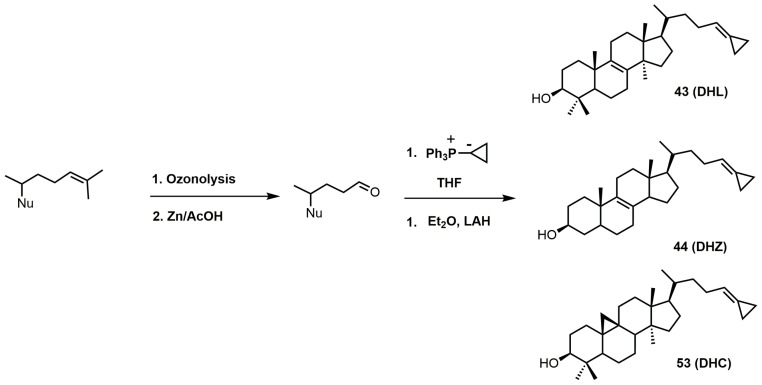
Synthesis of cyclopropylidine sterol derivatives. Nu: sterol nucleus (cycloartenol, lanosterol, or zymosterol).

The 26,27-dehydrosterols can be prepared in a few steps starting with the 3-acetylated sterol (Figure 25). The 24–25 double bond of the corresponding sterol is transformed into an aldehyde by ozonolysis with a reductive workup [102]. The 26,27-cyclopropylidine moiety is installed via a Wittig reaction using the desired sterol aldehyde and the phosphorus ylide formed from cyclopropyltriphenylphosphonium bromide and butyllithium [102]. The acetate group was easily removed with LAH.

**Figure 26:**
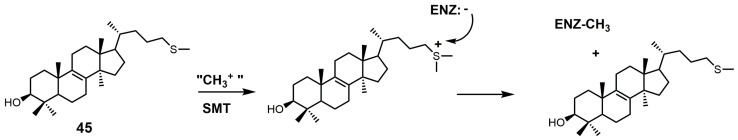
Proposed inhibitory mechanism of SMT with 25-thialanosterol (adapted from [4]).

Another class of 24-SMT inhibitors comprises sulfur-containing sterols such as 25-thialanosterol (**45**) and 25-thialanosterol iodide (**46**) (Figure 19) [103]. 24-SMT from *C. albicans* was irreversibly inhibited with 25-thialanosterol with a *K_i_* value of 5 μM and an apparent *k*_inact_ value of 0.013 min^−1^, while the corresponding sulfonium salt was a reversible transition-state inhibitor with a *K_i_* value of 20 nM [103]. 24-Thialanosterol was observed to inhibit *C. albicans* 24-SMT with an IC_50_ value of 20 μM [3]. 25-Thialanosterol iodide has a *K_i_* value of 86 nM against *T. brucei* 24-SMT and an ED_50_ value of 2 μM against both the procyclic and bloodstream forms of the *T. brucei* parasite [2]. 24-SMT can methylate the 25-sulfur atom of 25-thialanosterol to form a sulfonium cation that can act as a methyl donor to irreversibly methylate SMT (Figure 26) [4].

**Figure 27:**
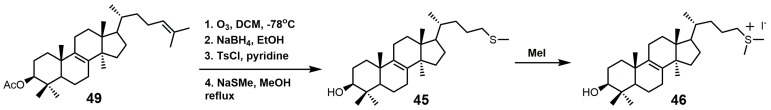
Synthesis of 25-thialanosterol and 25-thialanosterol salt.

The synthesis of 25-thialanosterol (**45**) and 25-thialanosterol iodide (**46**) is outlined in Figure 27 [103]. The synthesis started with ozonolysis of acetylated lanosterol (**49**) to yield 3-acetoxylanosta-8,24-dienol-26-al. 3-Acetoxylanosta-8,24-dienol-26-al was then reduced with sodium borohydride to yield an alcohol that was activated as a tosylate. Methanethiolate then displaced the tosylate group to yield 25-thialanosterol (**45**). Treatment of 25-thialansterol with methyl iodide yielded 25-thialanosterol iodide (**46**).

**Figure 28:**
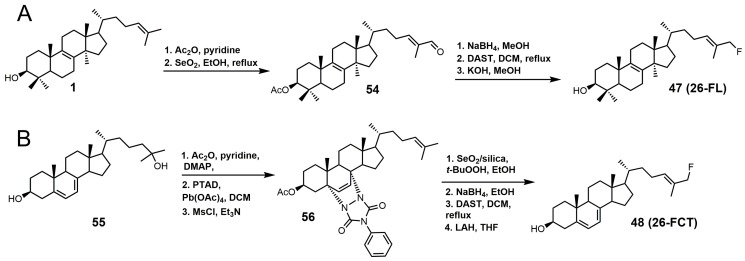
(**A**) Synthesis of 26-fluorolanosterol (26-FL) (**47**); (**B**) synthesis of 26-fluorocholesta-5,7,24-trienol (26-FCT) (**48**).

26-Fluorolanosterol (26-FL; **47**) is metabolically converted by *T. brucei* into a fluorinated substrate (26-fluorozymosterol) that irreversibly binds to 24-SMT and inhibits ergosterol biosynthesis and growth of both the procyclic and bloodstream forms of *T. brucei* [5]. *T. brucei* cell-based studies were conducted with 26-FL, and IC_50_ values for the procyclic and bloodstream forms were 3 and 16 μM, respectively, while 26-FL had no effect on HEK cell growth at up to 100 μM [5]. In order to further investigate the preferred substrate for *Tb*SMT, 26-fluorocholesta-5,7,24-trienol (26-FCT) was synthesized (Figure 28). 26-FCT was synthesized instead of 26-fluorozymosterol because of the difficulty in obtaining sufficient amounts of zymosterol to enable synthetic manipulation. 26-FCT was observed to be a competitive inhibitor of 24-SMT with respect to zymosterol and exhibited a *K_i_* value of 75 μM [5]. 26-FCT was confirmed to be a metabolite of 26-FL when metabolized by the procyclic form of *T. brucei* by GCMS with an authentic sample of 26-FCT [5]. 26-FCT has an excellent partition ration of 1.08, comparing favorably with eflornithine, which has a partition ratio of 3.3 against L-ornithine decarboxylase [5]. Both 26-FL and 26-FCT exhibit desirable drug characteristics with good specificity and low toxicity, and they might be useful in treating sleeping sickness or other protozoan infections [5]. When 26-FL is methylated by *Tb*SMT, two carbocations can form as short-lived intermediates that can be transformed into various turnover products (Figure 27) [5]. The kill product is where the 24-SMT enzyme has a prosthetic group attached, whereby SMT is irreversibly inhibited. 

26-FL was synthesized by the method used to synthesize 26-fluorocycloarentol (Figure 28A) [104]. The 3-hydroxy group of lanosterol was acetylated, and then the 26 methyl group was oxidized to an aldehyde (**54**) with selenium dioxide. The aldehyde was then reduced to the alcohol with sodium borohydride, and then the fluorine atom was installed via DAST. The acetate group was removed by potassium hydroxide in methanol to yield 26-FL.

The synthesis of 26-FCT started with compound **55**, which can be synthesized in eight steps starting with ergosterol (Figure 28B) [105]. Compound **55** was then acetylated, the Δ5 and Δ7 double bonds were protected with 4-phenyl-1,2,4-triazoline-3,5-dione (PTAD), and the Δ24–25 double bond was successfully installed with the use of mesyl chloride and triethylamine to yield compound **56**. The last four steps in the synthesis of 25-FCT were similar to the last four steps in the synthesis of 26-FL, except that global deprotection in the last step was accomplished with LAH.

## 5. Bifunctional 24-SMT and SDM Inhibitors

**Figure 29:**
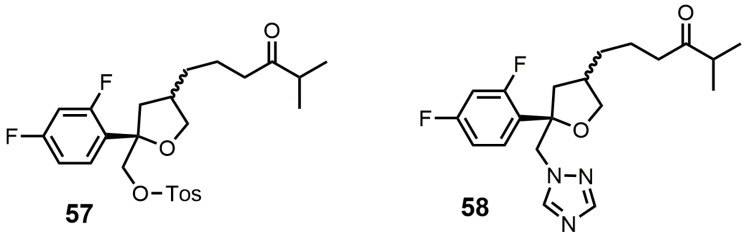
Structures of compounds designed to be bifunctional SDM and sterol C24-methyltransferase (24-SMT) inhibitors.

Compounds **57** and **58** (Figure 29) were designed to be dual functional SDM and 24-SMT inhibitors [106]. These compounds completely inhibited SDM from rat liver microsomes at 10 μM and showed reasonable in vitro potencies against *C. albicans*, *C. neoformans*, *A. fumigatus*, *T. mentagrophytes*, *Candida. pseudotropicalis,* and *Candida. krusei* with MIC values ranging from <0.1 to 50 mg/mL [106]. Compounds **57** and **58** were then tested in vivo against a murine candidiasis antifungal model, and unfortunately both compounds were ineffective against the induced infection. No explanation was provided as to why these compounds were ineffective in the in vivo model.

## 6. Conclusions

The inhibition of ergosterol biosynthesis in fungi and parasitic protozoa via the inhibition of SDM or 24-SMT with small molecules has been shown to be effective. Azole antifungals that target SDM have already been approved for the treatment of various fungal infections; however, they have not been officially approved to treat protozoan infections, despite various azoles advancing to clinical trials. Posaconazole was investigated in two phase II clinical trials as a possible agent to treat Chagas disease, and on the basis of the results provided thus far, it is unlikely that posaconazole will progress to phase III clinical trials [107,108,109]. The inhibition of fungal and protozoan 24-SMT with rationally designed molecules that are specific, selective, and non-toxic to humans have the potential to be used as the next generation of drugs to treat fungal infections or neglected tropical diseases that are demonstrating resistance against current therapies. It is anticipated that in the near future, both fungal and protozoan infections will be treated with a combination therapy that utilizes the cocurrent administration of a SDM and a 24-SMT inhibitor.
